# Factors influencing health-related quality of life in adolescent girls: a path analysis using a multi-mediation model

**DOI:** 10.1186/s12955-022-01954-6

**Published:** 2022-03-24

**Authors:** Hyunjeong Shin, Songi Jeon, Inhae Cho

**Affiliations:** 1 College of Nursing, Korea University, 145 Anam-ro, Sungbuk-gu, Seoul, 02841 Korea; 2 Department of Nursing, Catholic Kwandong University, Gangneung, Korea

**Keywords:** Adolescent, Depression, Dietary habits, Menstruation, Quality of life, Sleep, Social support

## Abstract

**Background:**

Recent studies have reported gender differences in adolescents’ health-related quality of life (HRQOL), with females scoring significantly lower than males. Researchers have identified the female puberty process as one of the causes of the differences in HRQOL between male and female adolescents. This study examines mechanisms of how social support, dietary habits, sleep quality, and depression contribute to predicting HRQOL in relation to menstrual health among adolescent girls.

**Methods:**

A cross-sectional study was conducted with 295 students recruited from middle and high schools in Korea using a self-report questionnaire. A multi-mediation model was constructed based on previous literature and tested using path analysis with AMOS, version 21.0.

**Results:**

The study results showed that menstrual health, social support, sleep quality, dietary habits, and depression had significant effects on HRQOL. Both sleep quality and depression had significant direct effects on menstrual health. Dietary habits, social support, sleep quality, and depression had significant indirect effects on HRQOL, mediated through menstrual health. According to serial mediation analysis, the path from social support to HRQOL via dietary habits → sleep quality → depression → menstrual health → HRQOL was significant. However, mediation models including the path of dietary habits → depression were not supported. The study variables explained 57% of the total variance for HRQOL.

**Conclusions:**

The findings suggest menstrual health is an important factor that mediates the effects of eating, sleeping, psychological health, and social support on HRQOL. Early complaints about sleep disorders and depressive symptoms with poor dietary habits could be an ominous sign for adolescent girls at high risk of menstrual problems and lower HRQOL. Empirical evidence from this study suggests the need to develop and test interventions addressing multiple modifiable behavioral and psychosocial factors to improve HRQOL in adolescent girls. Interventions or supportive systems that aim to improve eating habits and sleep quality thereby achieving a healthier lifestyle need to be developed and incorporated into school health services.

## Background

Health-related quality of life (HRQOL) is increasingly perceived as a central focus in health research [1]. Recent studies have reported gender differences in adolescents’ HRQOL, with females scoring significantly lower than males [2, 3]. Researchers have identified the female puberty process as one of the causes of the differences [2].

Menarche and menstruation with hormonal changes are remarkable features during puberty and adolescence in females. Menstrual problems, such as heavy menstrual bleeding, menstrual pain, abnormal cycle length, and an irregular menstrual cycle, are common among adolescent girls [4]. These problems are associated with poor academic performance and limitations in daily activities, which lead to decreased HRQOL in adolescent girls [5].

Past research also suggests that social support, dietary habits, sleep quality, and depression are interrelated and influence menstrual health and HRQOL in adolescent girls. Social support is associated with health behaviors and HRQOL. It influences health behaviors through the mechanism of improving the ability to access new information and developing interpersonal exchanges that provide encouragement to engage in healthy lifestyle practices including dietary habits or exercises [6, 7]. Dietary intake such as much caffeine or deficiency of calcium and magnesium plays an important role in the occurrence of menstrual problems in relation to symptoms of depression and sleep problems [8–10]. Sleep quality and depressive feelings have been reported to be related to menstrual health and reported as predictors of HRQOL [11, 12].

Previous studies, however, have focused on the patterns of the relationship between menstrual health and its affecting factors such as diet, sleep, and mental health [13, 14] or on how menstrual problems influence HRQOL [15]. However, none of the studies has not researched these variables simultaneously. Further studies are warranted to explore the interrelationships among them. Understanding how different factors contribute to predicting HRQOL and how menstrual health operates in this process will help health professionals to be more targeted and effective in promoting HRQOL in female adolescents.

### Hypotheses development

Social support has been recognized as an important predictor of health and HRQOL. A school-based survey reported that supportive social relationships from family members, friends, and schoolmates had a positive impact on HRQOL in adolescents [6]. In a study among young women, Alonso and Coe [16] reported that women with more disruptions in their social networks experienced more menstrual symptoms than did women with stable support. However, rather than a direct influence on menstrual symptoms, the authors explained that losing a valued personal relationship may reduce a woman’s capacity to manage the painful symptoms and actually increase neuroendocrinological and proinflammatory physiology related to dysmenorrhea. Social relationships also have been reported to be associated with depression and sleep quality [17]. A study with Korean adolescents found that social support affects premenstrual symptoms through psychological factors such as depression and stress [18]. More parental support was also reported to be linked to better sleep quality in adolescents [19].

In addition, in adolescent groups, social support was found to be an affecting factor for their lifestyle habits which are predictors of menstrual health [7, 20]. A systematic review reported that parental support has an influence on an adolescent healthy diet, meaning positive relationships between social support and dietary habits in adolescents [7]. Thus, we hypothesized that:

- H1: Higher social support is associated with higher HRQOL.
- H2: Higher social support is associated with lower depression.
- H3: Higher social support is associated with better sleep quality.
- H4: Higher social support is associated with healthier dietary habits.

Studies on adolescent health have reported relationships between menstrual health and the lifestyle factors, such as sleep quality and dietary habits [4, 14]. Sleep quality has been reported as one of the potential risk factors for menstrual pain and girls’ HRQOL [11, 12]. A previous study reported that poor sleep quality, especially less sleep, is related to primary dysmenorrhea in adolescents [11]. Other studies reported sleep quality as a significant affecting factor for menstrual irregularities [14]. Sleep quality is also associated with adolescents’ HRQOL and their own perception of physical and mental health. Paiva et al. [21] reported that HRQOL was lower in adolescents with sleep deprivation showing girls had significantly more health complaints than boys. Both insomnia and a short duration of sleep were found to increase the risk of depression in adolescents in several epidemiological studies [22, 23]. Thus, we hypothesized that:

- H5: Poor sleep quality is associated with lower HRQOL.
- H6: Poor sleep quality is associated with higher depression.
- H7: Poor sleep quality is associated with poor menstrual health.

Other lifestyle variables, such as poor dietary patterns have also been reported to be risk factors for menstrual problems [11]. Previous studies suggested that breakfast skipping affects adolescents’ dietary patterns, reporting adolescents skipping breakfast had more often sugar-sweetened foods [24]. A high intake of junk food and soft drink was reported to be associated with poor sleep quality in Korean adolescents [8]. Dietary patterns are also associated with depression. Studies reported that a lower intake of calcium, potassium, vitamin C, vitamin D, and proteins was related to depressive symptoms and sleep disturbances [9, 10]. A systematic review also identified the impact of diet on mental health, reporting healthy dietary patterns or consumption of a high-quality diet are related to the lower level of depression [25]. Thus, we hypothesized that:

- H8: Healthier dietary habits are associated with better sleep quality.
- H9: Healthier dietary habits are associated with lower depression.

There is growing evidence of an association between menstrual cycle dysfunction and mental health problems in adolescents and young women [12]. Studies have reported that girls with more depressive symptoms were at greater risk for experiencing menstrual symptoms and lower levels of quality of life (QOL) [26]. A study among Korean adolescents reported positive associations between depressive symptoms and menstrual cycle irregularity [4]. Several studies reported direct effects of depressive symptoms on QOL in adolescent populations [27, 28]. Thus, we hypothesized that:

- H10: Higher depression is associated with poor menstrual health.
- H11: Higher depression is associated with lower HRQOL.

Past research has studied the relationship between QOL and several types of menstrual problems, such as dysmenorrhea, heavy menstrual bleeding, and premenstrual syndrome (PMS). A study of women with primary dysmenorrhea confirmed that menstrual cramping pain substantially reduces HRQOL [15]. QOL was poorer in women who complained of the irregular menstrual cycle than in those who did not have such complaints [29]. Several studies have shown that women’s HRQOL is negatively affected by menstrual dysfunction [30, 31]. Research has found a significant association between PMS scores and QOL [32]. Thus, we hypothesized that:

- H12: Better menstrual health is associated with higher HRQOL.

**Fig. 1:**
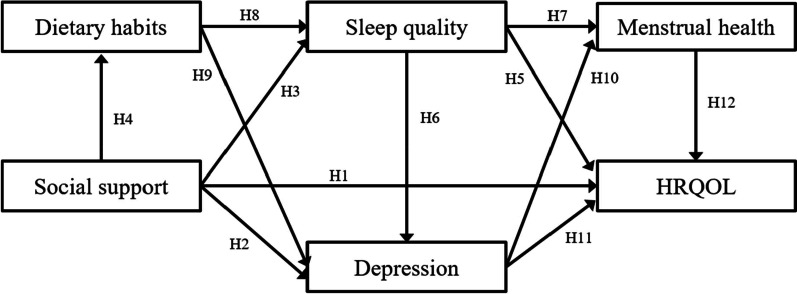
Hypothesized relations. *Note*. HRQOL: health-related quality of life

Drawing on these hypotheses, Fig. 1 depicts the hypothetical path model proposed. Based on Fig. 1, increasing social support in adolescent girls can make their dietary habits and sleep quality better, reduce depressive feelings, and improve their menstrual health and HRQOL. Thus, we hypothesized that:

H13: Dietary habits, sleep quality, depression, and menstrual health have significant mediating effects in the relationship between social support and HRQOL.

Collectively, past research suggests that the variables of dietary habits, social support, sleep quality, and depression are interwoven and influence adolescent girls’ menstrual health and HRQOL directly or indirectly; and menstrual health affects their HRQOL in adolescent girls. Accordingly, the present study tested a model specifying these multi-mediation pathways.

### Study purpose

The purpose of this study was to test a hypothetical path model estimating the direct or indirect influence of dietary habits, social support, sleep quality, depression, and menstrual health on HRQOL in adolescent girls (Fig. 1).

## Methods

### Participants

A cross-sectional survey was conducted from six middle schools and three high schools in Korea between December 2015 and March 2016. The schools were located in Seoul and the metropolitan area and they were selected by convenience sampling methods. The study was approved by the University Institutional Review Board and ethical committee (IRB 15215A1). All participants and their parents were informed about the study and given information about the right to withdraw from it without any penalties. Contact information and the questionnaire were provided, and all questionnaire items were answered anonymously. All participants and their parents agreed to participate in the study and signed the informed assent form and parental consent form. Eligibility criteria included: (1) students who were in middle schools or high schools (there was no limitation in female students’ age), (2) students whose time since menarche was more than 12 months (because the onset and around of menarche is often associated with problems of irregular menstruation, excessive bleeding, and dysmenorrhea) [33], (3) students who were not pregnant, and (4) female adolescents who could read and understand Korean.

The estimated sample size was 161, based on an effect size of 0.10, αof 0.01, power of 0.95, and a total number of predictors of 5 using G*Power. A total of 295 students was recruited using a convenient sampling method, and all of them participated in the study. Among the 295 students enrolled in the study, 291 students (98.6%) completed all of the questionnaires and were included in the analyses for the present study. The sample size of 291 also exceeded the recommended minimum of 250 cases for bootstrap tests of indirect effects in a path analysis [34].

### Procedures

Data were collected by two trained research assistants and school nurses from December 2015 to February 2016 using self-report questionnaires. After obtaining permission from schools, research assistants or school nurses explained the study and eligibility criteria to students during lunchtimes or break times. Students who wanted to participate in the study visited school health rooms. Research assistants or school nurses informed the students about the study in the room. Data were collected from the students who signed on the informed assent form. Among the informed students, those who were less than 18 years old were given a consent form and a study leaflet with the questionnaire. They showed the questionnaire and the leaflet to their parents and got permission by having their parents sign parental consent forms. Students who got permission from their parents completed the questionnaire after signing the informed assent form.

### Measures

#### Health-related quality of life

HRQOL was measured by the PedsQL 4.0 Generic Core Scale [35]. It is a multidimensional instrument measuring physical, emotional, social, and school functioning in children and adolescents and has been translated into numerous languages [36]. Choi’s [36] Korean version was used in the present study. It is a 5-point response scale (0 = *never a problem*, 4 = *almost always a problem*). All items were reverse-scored and transformed to a 0 to 100 scale (0 = 100, 1 = 75, 2 = 50, 3 = 25, 4 = 0) so that higher scores indicate better HRQOL. Cronbach’s alpha coefficient was 0.93 in Choi’s study [36] among Korean adolescents and 0.91 in the present study.

#### Menstrual health

Menstrual health was measured by the Menstrual Health Instrument (MHI) developed by Shin and her colleagues [37]. It uses a 4-point Likert-type scale and consists of 29 items assessing affective symptoms, somatic symptoms and school life, daily habits for menstrual health, menstrual cycle characteristics, and attitudes toward and perceptions of menstruation. Higher scores indicate better menstrual health status. Cronbach’s alpha coefficient was 0.91 when it was developed among Korean adolescent girls [37] and 0.92 in this study.

#### Depression

Depression was measured with the Center for Epidemiological Studies Depression Scale (CES-D), developed by Radloff [38]. We used Chon and Rhee’s [39] Korean version. Participants were asked how often they have had depressive symptoms in the past week across 20 items. A 4-point Likert-type scale was used, and higher scores indicate higher levels of depressive symptoms. Cronbach’s alpha coefficient was 0.89 in Chon and Rhee’s [39] study and 0.90 in the current study.

#### Sleep quality

To measure sleep quality, we used a Korean version of the Sleep Quality Index [40], the items which were taken from the Pittsburgh Sleep Quality Index [41]. For this study, we have modified the Korean version of the Sleep Quality Index to be adequate for Korean middle and high school students (i.e., the item of ‘how often have you had trouble staying awake while driving or engaging in social activity’ was deleted). The reduced and modified 15-item scale measures the level of sleep disturbances and is rated on a 4-point response format. Higher scores indicate poorer sleep quality with more severe sleep disturbances. Cronbach’s alpha coefficient was 0.74 when it was translated into Korean [40] and 0.77 in this study.

#### Dietary habits

Dietary habits were measured with 9 items from the Korean Youth Risk Behavior Web-Based Survey [42], which is conducted annually by the Korean Center for Disease Control. Items were about food intake containing caffeine, sugar, calcium, fruits and vegetables, and fast foods. The items measured the number of food intake during the past one week using a 7-point Likert scale. The more the respondents ate caffeine, sugar, and fast foods, the lower the scores were given. The more they ate calcium, fruits, and vegetables, the higher the scores were given. Thus, higher scores indicate healthier dietary habits.

#### Social support

Social support was measured by the Perceived Social Support Scale (PSSS) developed by Han and Yoo [43]. It is a 5-point Likert scale and consists of 24 items regarding perceived support from family, school teachers, and friends. Higher scores indicate higher levels of social support. Cronbach’s alpha coefficient was 0.92 in the past study among Korean adolescents [43] and 0.93 in the current study.

### Statistical analysis

SPSS for Windows, version 21.0 was used to calculate the descriptive statistics for summarizing the demographic characteristics of the participants and the correlation analyses between study variables. The normality of the study variables was tested with the Kolmogorov–Smirnov test, verifying normal distributions of the six variables (W = 0.14–0.20, *p* = 0.07–0.20).

The path model testing was conducted using AMOS, version 21.0. Before testing the model fit, tolerance and variance inflation factor (VIF) were computed using the SPSS program to detect multi-collinearity problems. The model fit was examined using the following goodness-of-fit indices: the Chi-square value (desired *p*-value > 0.05), the goodness-of-fit index (GFI: desired value > 0.95), normed fit index (NFI: desired value > 0.95), comparative fit index (CFI: desired value > 0.95), Tucker-Lewis index (TLI: desired value > 0.95), root mean square error of approximation (RMSEA: desired value < 0.06), and standardized root mean square residual (SRMR: desired value < 0.08). [44].

To test whether there were serial multiple mediation effects of dietary habits, sleep quality, depression, and menstrual health between social support and HRQOL, a serial multiple mediation analysis using phantom variables was conducted. In the present study, we set the bootstrap confidence interval (CI) at 95%, and the number of bootstrap samples was 2,000. If the 95% CI does not contain zero, it indicated that the mediating effect was significant.

## Results

### Characteristics of the participants

The mean age of the participants was 16.35 (SD = 1.53) with a range of 14–19 years. Half of the participants (50.5%) were high school students. The mean BMI was 20.25 (SD = 2.61). More than one-third of the participants (37.5%) skipped breakfast more than four days per week. The mean age at menarche was 13.30 (SD = 1.07) with a range of 10–16 years. The average usual menstrual cycle length was 32.08 days (SD = 14.14) and the duration was 6.07 days (SD = 1.49). Of the participants, 10.0% had visited clinics for menstrual problems. The demographic and menstruation-related characteristics of the participants are presented in Table 1.

**Table 1:** Demographic and menstruation-related characteristics of the participants (N = 291)

Variables	N	%	Mean	SD
Age (years)			16.35	1.53
School				
Middle school students	144	49.5		
High school students	147	50.5		
Body weight (kg)			52.03	8.27
Height (cm)			160.07	5.39
Body mass index			20.25	2.61
< 18.5	64	22.0		
18.5 to < 25.0	195	67.0		
25.0 to < 30.0	11	3.8		
≥ 30	2	0.7		
Missing	19	6.5		
Breakfast skipping				
More than 4 days/week	109	37.5		
Less than 3 days/week	182	62.5		
Age at menarche (years)			13.30	1.07
Usual menstruation cycle length (days)			32.08	14.14
Usual duration of menstruation (days)			6.07	1.49
Menstrual pain intensity (possible range: 1–10)			4.33	2.86
Experiences of visiting clinic for menstrual problems				
Yes	29	10.0		
No	262	90.0		

### Relationships between study variables

HRQOL was significantly correlated with dietary habits (*r* = 0.34, *p* < 0.01), sleep quality (*r* = − 0.50, *p* < 0.01), social support (*r* = 0.48, *p* < 0.01), depression (*r* = − 0.61, *p* < 0.01), and menstrual health (*r* = 0.53, *p* < 0.01).

Menstrual health was significantly correlated with dietary habits (*r* = 0.21, *p* < 0.01), sleep quality (*r* = − 0.50, *p* < 0.01), social support (*r* = 0.22, *p* < 0.01), depression (*r* = − 0.52, *p* < 0.01), and HRQOL (*r* = 0.53, *p* < 0.01). Correlation coefficients between study variables are presented in Table 2.

**Table 2:** Correlations among the study variables (N = 291)

Variables	Possible range	Mean	SD	1	2	3	4	5	6
1. Dietary habits	9–63	49.41	6.06	1.00					
2. Sleep quality	0–45	10.64	5.78	− 0.34**	1.00				
3. Social support	24–120	91.55	13.30	0.34**	− 0.31**	1.00			
4. Depression	20–80	34.69	9.00	− 0.34**	0.53**	− 0.51**	1.00		
5. Menstrual health	29–116	78.56	15.11	0.21**	− 0.50**	0.22**	− 0.52**	1.00	
6. HRQOL	0–100	80.17	13.38	0.34**	− 0.50**	0.48**	− 0.61**	0.53**	1.00

*HRQOL* Health-related quality of life

**p < 0.01

### Fitness of the path model

In the current study, the VIF was a range of 1.25–1.92, which did not exceed the standard value of 10, and tolerance was a range of 0.66–0.80, which was larger than 0.10, indicating no multi-collinearity between study variables.

Testing of the hypothetical path model showed that the model fit was good (χ^2^ [3, 291] = 3.52, *p* = 0.32, GFI = 0.99, NFI = 0.99, CFI = 0.99, TLI = 0.99, RMSEA = 0.02 [90% of confidence interval = 0.00 (0 < Lowest level of CI < 0.01) ~ 0.11], SRMR = 0.02). Among the 12 paths in the model, one (dietary habits → depression) was not statistically significant.

### Direct effects of study variables on HRQOL

**Fig. 2:**
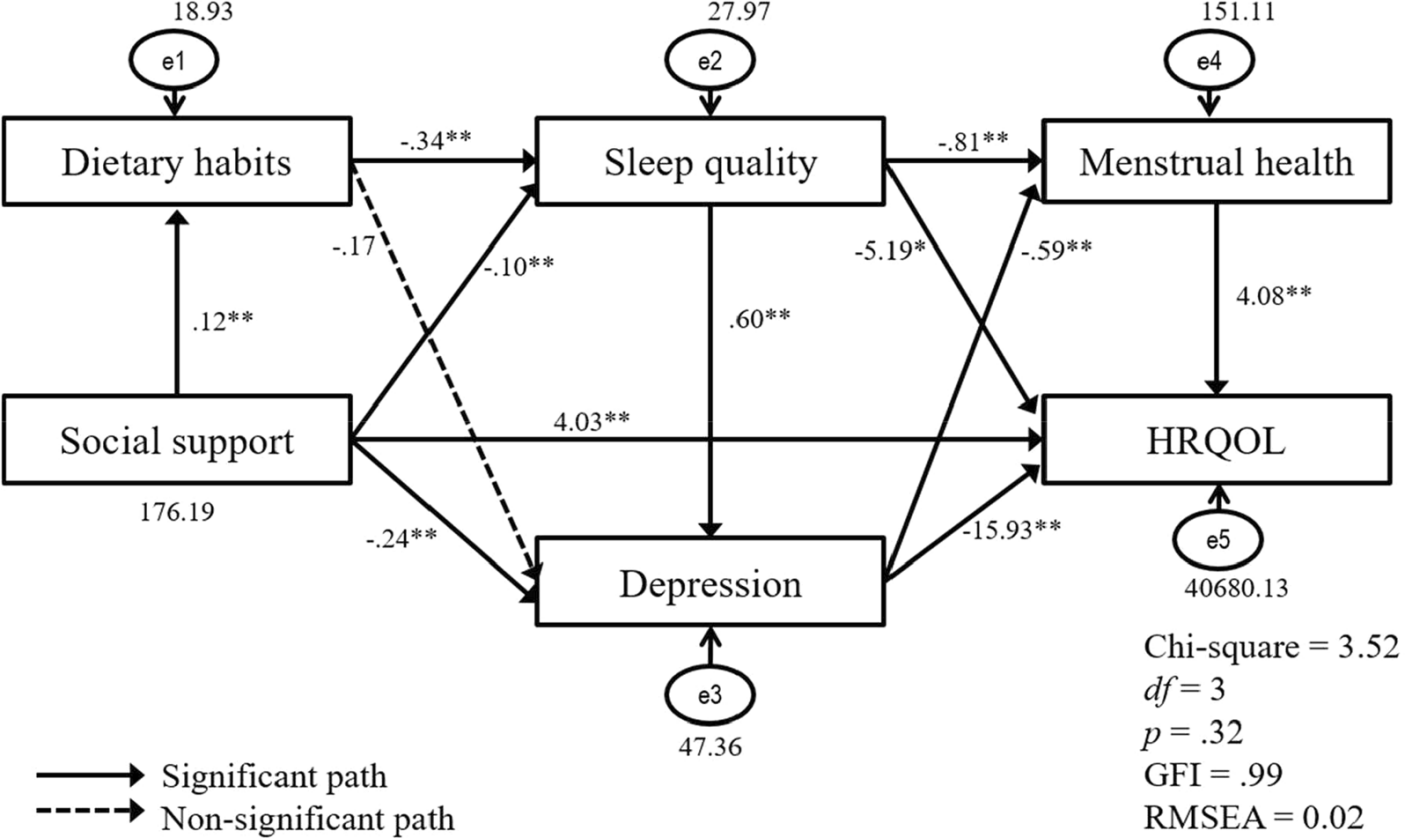
Result of the path model testing. *Note*: *HRQOL* health-related quality of life; * *p* < .0.05, ** *p* < .0.01; *GFI* goodness-of-fit index, *RMSEA* root mean square error of approximation

The results of the analysis of effects showed that social support (b = 4.03, *p* < 0.01) and menstrual health (b = 4.08, *p* < 0.01) had significant direct effects on HRQOL in a positive direction. Sleep quality (b = − 5.19, *p* < 0.05) and depression (b = − 15.93, *p* < 0.01) were also directly associated with HRQOL but in a negative direction (Fig. 2).

### Indirect effects of study variables on HRQOL

As shown in Table 3, the summative indirect effects of dietary habits, social support, sleep quality, and depression on HRQOL were significant. Dietary habits (b = 9.77, *p* < 0.05) and social support (b = 7.44, *p* < 0.01) had positive indirect effects on HRQOL. Sleep quality (b = − 14.27, *p* < 0.01) and depression (b = − 2.41, *p* < 0.01) also showed significant indirect influence on HRQOL but in a negative direction. The five variables (social support, dietary habits, sleep quality, depression, and menstrual health) explained 57% of the total variance for HRQOL. The unstandardized and standardized direct, indirect, and total effects of the independent variables on HRQOL are shown in Table 3.

**Table 3:** Direct, indirect, and total effect coefficients and SMC of the variables

Endogenous variables	Predicting variables	Direct effect			(summative) Indirect effect			Total effect			SMC
		b	95% CI	β	b	95% CI	β	b	95% CI	β	
Dietary habits	Social support	0.12**	0.09, 0.15	0.34**				0.12**	0.09, 0.15	0.34**	0.11
Sleep quality	Dietary habits	− 0.34*	− 0.47, − 0.20	− 0.27*				− 0.34*	− 0.47, − 0.20	− 0.27*	0.16
	Social support	− 0.10**	− 0.13, − 0.06	− 0.22**	− 0.04*	− 0.06, − 0.02	− 0.09*	− 0.14**	− 0.17, − 0.09	− 0.31**	
Depression	Dietary habits	− 0.18	− 0.34, 0.01	− 0.09	− 0.20*	− 0.29, − 0.12	− 0.10*	− 0.38*	− 0.56, − 0.20	− 0.19*	0.41
	Social support	− 0.24*	− 0.32, − 0.17	− 0.36*	− 0.10**	− 0.14, − 0.07	− 0.15**	− 0.34*	− 0.41, − 0.27	− 0.51*	
	Sleep quality	0.60**	0.48, 0.73	0.38**				0.60**	0.48, 0.73	0.38**	
Menstrual health	Dietary habits				0.50*	0.28, 0.72	0.15*	0.50*	0.28, 0.72	0.15*	0.34
	Social support				0.31**	0.24, 0.38	0.28**	0.31**	0.24, 0.38	0.50**	
	Sleep quality	− 0.81*	− 1.06, − 0.58	− 0.31*	− 0.35**	− 0.48, − 0.25	− 0.14**	− 1.17*	− 1.40, − 0.96	− 0.45*	
	Depression	− 0.59**	− 0.75, − 0.45	− 0.35**				− 0.59**	− 0.75, − 0.45	− 0.35**	
HRQOL	Dietary habits				9.77*	5.56, 14.23	0.15*	9.77*	5.56, 14.23	0.15*	0.57
	Social support	4.03*	2.08, 5.61	0.17*	7.44**	5.80, 9.36	0.35*	11.47*	9.22, 13.52	0.50*	
	Sleep quality	− 5.19*	− 8.93, − 1.12	− 0.10	− 14.27**	− 18.67, − 10.95	− 0.27*	− 19.46**	− 23.93, − 15.02	− 0.37**	
	Depression	− 15.93*	− 19.35, − 13.01	− 0.50*	− 2.41**	− 3.60, − 1.39	− 0.07**	− 18.34*	− 21.79, − 15.72	− 0.54*	
	Menstrual health	4.08*	2.56, 5.58	0.20*				4.08*	2.56, 5.58	0.20*	

*HRQOL* Health-related quality of life, *b* unstandardized coefficients, *β* standardized coefficients, *SMC* Squared Multiple Correlations

*p < 0.05; **p < 0.01

### Mediating effects of dietary habits, sleep quality, depression, and menstrual health between social support and HRQOL

To test the mediating effect of dietary habits, sleep quality, depression, and menstrual health in the relationship between social support and HRQOL, a serial multiple mediation analysis was conducted. Although the summative indirect effect of social support on HRQOL was significant in Table 3, the individual indirect effect was different according to the detailed path. The results are shown in Table 4.

**Table 4:** Indirect effects between social support and HRQOL for serial mediation

Paths	b	SE	β
via sleep quality → HRQOL	0.49*	0.27	0.02
via depression → HRQOL	3.87*	0.86	0.17
via sleep quality → depression → HRQOL	0.90**	0.27	0.04
via dietary habits → depression → HRQOL	0.32	0.23	0.01
via dietary habits → sleep quality → HRQOL	0.21*	0.12	0.01
via dietary habits → sleep quality → depression → HRQOL	0.38*	0.13	0.02
via sleep quality → menstrual health → HRQOL	0.31**	0.12	0.01
via depression → menstrual health → HRQOL	0.59**	0.18	0.03
via sleep quality → depression → menstrual health → HRQOL	0.14**	0.05	0.01
via dietary habits → sleep quality → menstrual health → HRQOL	0.13*	0.06	0.01
via dietary habits → depression → menstrual health → HRQOL	0.05	0.04	0.01
via dietary habits → sleep quality → depression → menstrual health → HRQOL	0.06**	0.02	0.01
Total indirect effect	7.44**	1.05	0.35

*HRQOL* Health-related quality of life, *b* unstandardized coefficients, *β* standardized coefficients, *SE* standard error

*p < 0.05; **p < 0.01

Social support was associated with HRQOL through depression (b = 3.87, *p* < 0.05), and it was serially associated with HRQOL through depression and menstrual health (b = 0.59, *p* < 0.01). Social support was also serially associated with HRQOL through sleep quality and menstrual health (b = 0.31, *p* < 0.01). Moreover, the indirect effect of social support through the three-mediator pathways of sleep quality, depression, and menstrual health was significant (b = 0.14, *p* < 0.01). Social support was also serially associated with HRQOL through dietary habits, sleep quality, and menstrual health (b = 0.13, *p* < 0.05). In addition, the indirect effect of social support through the four-mediator pathways of dietary habits, sleep quality, depression, and menstrual health was significant (b = 0.06, *p* < 0.01).

However, the path of social support → dietary habits → depression → HRQOL (b = 0.32, *p* = 0.12) and the path of social support → dietary habits → depression → menstrual health → HRQOL (b = 0.05, *p* = 0.08) were not significant.

## Discussion

The mediation model gives the answers to questions of how an effect takes place [45]. The mediation process outlined in this study shows how social support, dietary habits, sleep quality, and depression influence female adolescents’ HRQOL in relation to menstrual health. In this study, the path from social support to HRQOL via dietary habits → sleep quality → depression → menstrual health → HRQOL was significant. The study findings suggest that adolescents who have more support from their families or schools might have better dietary habits, which leads to them having better sleep quality. The adolescents who sleep well might feel less depressed and experience fewer menstrual symptoms, which leads to better HRQOL. Current findings reminded that health care professionals should be conscious of potentially co-existing sleep and depressive symptoms when menstrual problems were reported, and it leads to a decline in HRQOL in adolescent girls. Early complaints about sleep disorders and depressive symptoms with poor dietary habits could be an ominous sign for adolescent girls at high risk of menstrual problems and lower HRQOL.

In this study, based on the estimates of the total effect (a summation of a direct effect and an indirect effect) in Table 3, we found that social support had great effects on both menstrual health and HRQOL. Adolescent perception of social support was associated directly with all study variables included in this study except menstrual health. The link between social support and the variables of dietary habits and sleep quality suggests that adolescents perceiving more support from their family and friends tend to have healthier and more desirable lifestyle habits. It must be noted that social support was also a direct predictor of HRQOL, revealing the importance of social ties on QOL and the well-being of adolescents [6].

Also, we found that sleep quality had great effects on both menstrual health and HRQOL. The total effect of sleep quality on HRQOL was significant but the indirect effects mediated through menstrual health or depression were much larger than the direct effects. Rather than through the sleep duration or quality itself, adolescent girls seem to evaluate their HRQOL through worsening psychological and physical symptom status with trouble sleeping. It might be attributable to chronically routinized shortened sleep duration in Korean adolescents. Due to the highly competitive educational system, Korean adolescents have an average of 6.5 h of sleep per night, which is substantially lower than the recommended 8–10 h per night [46]. Short sleep duration and sleep deprivation are usual daily life that every adolescent experiences in Korea; they seem to perceive their HRQOL is decreased not with less sleep itself but with symptoms developed from the poor sleep quality. Previous studies mentioned that poor sleep quality puts adolescents at risk for psychological problems and externalizing symptoms; thus, the literature emphasized the finding of factors that affect adolescents’ sleep quality and of developing interventions [47]. The present study extends the previous literature by utilizing data of Korean adolescents and confirms the negative role of poor sleep quality in externalizing symptoms across different cultures.

In addition to examining direct and summative indirect relationships between variables, we also tested whether there is a chain mediating role between social support and HRQOL. In the present study, we found that social support and dietary habits affected menstrual health indirectly through worsening sleep quality. Both sleep problems and depression are common in adolescence [23]. Previous studies have reported that sleep quality is a powerful predictor of menstrual health [11, 48]. Gagua and colleagues [11] reported that one of the most important risk factors of menstrual pain in adolescent populations was poor sleep quality, especially less sleep. Even though there are socio-cultural differences, the sleep duration of adolescents worldwide as well as in Korea is significantly less than the recommended nine to ten hours per night [11, 23]. Short sleep duration is strongly interrelated with depression, which has been mentioned as a potential risk factor for dysmenorrhea [12, 23]. Given that depression showed the greatest effect on HRQOL in adolescent girls in the current study, treating both conditions of sleep and depression concurrently seems warranted for improving menstrual health and HRQOL for adolescents who have a lack of social support.

According to serial mediation analysis, however, mediation models including the path of dietary habits → depression were not supported. The indirect effect of the social support on HRQOL via dietary habits and depression was not statistically significant, nor was the three-mediator indirect effect through dietary habits, depression, and menstrual health. Given that a significant zero-order correlation was observed between dietary habits and the variable of depression, it is interesting that non-significant path coefficients were found in the model. According to the study results, the direct effect of dietary habits on depression was not significant. Instead, dietary habits indirectly influenced depression through sleep quality. Although many studies have indicated a relationship between diet and mental health and have attempted to explain how diet and nutrition modulate mental health status [10, 49], the mechanisms are still not well understood [50]. The results of the current study suggest that sleep quality can be a mediator in the effect mechanism of diet on mental health. This also indicates that the depressive symptoms of those who have bad dietary habits stem from the extent to which they have simultaneously poor sleep quality.

In the current study, the independent variables—including social support, dietary habits, sleep quality, depression, and menstrual health—accounted for 57% of the total variance for HRQOL in adolescent girls. Undoubtedly there may be other variables contributing to adolescents’ HRQOL and menstrual health, such as hereditary factors or physical illnesses that might aggravate menstruation-related symptoms [51, 52]. Future research considering these variables may further explain menstrual health and HRQOL in adolescent girls and extend our knowledge regarding their predictive relationships.

Even though this is the first empirical study to examine how social support, dietary habits, sleep quality, and depression influence female adolescents’ HRQOL in relation to menstrual health, it has several limitations that need to be considered when interpreting the results. First, the use of convenience sampling may limit the generalizability of the study findings. The study sample may not be representative of all Korean adolescent girls. Second, this study used a cross-sectional design; thus, causality among study variables cannot be established. The directionality of relationships needs to be examined with longitudinal data. Third, we used self-reporting questionnaires to measure depression and menstrual health in this study rather than clinical diagnostic criteria by experts. Dietary habits were also measured by retrospective self-report of food consumption. Although the questionnaires used in this study had been validated in Korean adolescent populations [37, 53], further investigations adding more objective measures (e.g., daily food dietary information) may be helpful to gain a better understanding of the predictive relationships among the variables. Lastly, in the current study, 10% of the participants had experiences of visiting clinics for menstrual problems, but we did not consider the use of hormonal medications and their effects on menstrual patterns in the process of estimation of the model.

## Conclusion

The current study is an important step toward a better understanding of how social support, dietary habits, sleep quality, and depression interact with and influence HRQOL in relation to menstrual health in female adolescents. As supported by the current study, menstrual health is an important factor that mediates the effects of eating, sleeping, psychological health, and social support on HRQOL. Empirical evidence from this study suggests the need to develop and test interventions addressing multiple modifiable behavioral and psychosocial factors to improve HRQOL in adolescent girls; a comprehensive approach to improve HRQOL should consider the level of perceived social support, psychological characteristics, and their menstrual health status; interventions or supportive systems that aim to improve eating habits and sleep quality thereby to achieve a healthier lifestyle need to be developed and incorporated into school health services. Also, this was the first study exploring how social support, dietary habits, sleep quality, and depression influence adolescent girls’ HRQOL in relation to menstrual health. Further research is needed to generalize this model to other populations with different cultures.

## Data Availability

The data sets used and analyzed in the current study are available from the corresponding author on reasonable request.

## Abbreviations

HRQOL: Health-related quality of life
QOL: Quality of life
PMS: Premenstrual syndrome
GFI: Goodness-of-fit index
NFI: Normed fit index
CFI: Comparative fit index
TLI: Tucker-Lewis index
RMSEA: Root mean square error of approximation
CI: Confidence interval

## References

[1] Knox E, Muros JJ (2017). Association of lifestyle behaviors with self-esteem through health-related quality of life in Spanish adolescents. Eur J Pediatr.

[2] Loh DA, Moy FM, Zaharan NL, Mohamed Z (2015). Disparities in health-related quality of life among healthy adolescents in a developing country - the impact of gender, ethnicity, socio-economic status and weight status. Child Care Health Dev.

[3] Meade T, Dowswell E (2016). Adolescents' health-related quality of life (HRQoL) changes over time: a three year longitudinal study. Health Qual Life Out.

[4] Yu M, Han K, Nam GE (2017). The association between mental health and menstrual cycle irregularity among adolescent Korean girls. J Affect Disorders.

[5] Nur Azurah AG, Sanci L, Moore E, Grover S (2013). The quality of life of adolescents with menstrual problems. J Pediatr Adol Gynec.

[6] Gomes AC, Rebelo MAB, de Queiroz AC, de Queiroz Herkrath APC, Herkrath FJ, Rebelo Vieira JM (2020). Socioeconomic status, social support, oral health beliefs, psychosocial factors, health behaviours and health-related quality of life in adolescents. Qual Life Res.

[7] Cislak A, Safron M, Pratt M, Gaspar T, Luszczynska A (2011). Family-related predictors of body weight and weight-related behaviours among children and adolescents: A systematic umbrella review. Child Care Health Dev..

[8] Park S, Rim SJ, Lee JH (2018). Associations between dietary behaviours and perceived physical and mental health status among Korean adolescents. Nutr Diet.

[9] Jansen EC, Baylin A, Cantoral A, Rojo MMT, Burgess HJ, O’Brien LM (2020). Dietary patterns in relation to prospective sleep duration and timing among Mexico City adolescents. Nutrients.

[10] Zhan Y, Ma H, Feng Y, Wang Y, Wu S, Cai S (2020). Dietary patterns in relation to gestational depression and sleep disturbance in Chinese pregnant women. J Obstet Gynaecol Re.

[11] Gagua T, Tkeshelashvili B, Gagua D (2012). Primary dysmenorrhea: prevalence in adolescent population of Tbilisi, Georgia and risk factors. J Turkish-German Gyn Assoc.

[12] Mannix LK (2008). Menstrual-related pain conditions: Dysmenorrhea and migraine. J Womens Health.

[13] Bajalan Z, Moafi F, MoradiBaglooei M, Alimoradi Z (2019). Mental health and primary dysmenorrhea: a systematic review. J Psychosom Obst Gyn.

[14] Lim H-S, Kim T-H, Lee H-H, Park Y-H, Lee B-R, Park Y-J, Kim Y-S (2018). Fast food consumption alongside socioeconomic status, stress, exercise, and sleep duration are associated with menstrual irregularities in Korean adolescents: Korea National Health and Nutrition Examination Survey 2009–2013. Asia Pac J Clin Nutr.

[15] Wong CL (2018). Health-related quality of life among Chinese adolescent girls with dysmenorrhea. Reprod Health.

[16] Alonso C, Coe CL (2001). Disruptions of social relationships accentuate the association between emotional distress and menstrual pain in young women. Health Psychol.

[17] Recto P, Champion JD (2017). Psychosocial risk factors for perinatal depression among female adolescents: a systematic review. Issues Ment Health Nurs.

[18] Jeon JH, Hwang SK (2014). A structural equation modeling on premenstrual syndrome in adolescent girls. J Korean Acad Nurs.

[19] Tsai KM, Dahl RE, Irwin MR, Bower JE, McCreath H, Seeman TE (2018). The role of parental support and family stress in adolescent sleep. Child Dev.

[20] Jeon GE, Cha NH, Sok SR (2014). Factors influencing the dysmenorrheal among Korean adolescents in middle school. J Phys Ther Sci.

[21] Paiva T, Gaspar T, Matos M (2015). Sleep deprivation in adolescents: correlations with health complaints and health-related quality of life. Sleep Med.

[22] Merikanto I, Lahti T, Puusniekka R, Partonen T (2013). Late bedtimes weaken school performance and predispose adolescents to health hazards. Sleep Med.

[23] Sivertsen B, Harvey AG, Lundervold AJ, Hysing M (2014). Sleep problems and depression in adolescence: results from a large population-based study of Norwegian adolescents aged 16–18 years. Eur Child Adoles Psy.

[24] Smetanina N, Albaviciute E, Babinska V, Karinauskiene L, Albertsson-Wikland K, Petrauskiene A, Verkauskiene R (2015). Prevalence of overweight/obesity in relation to dietary habits and lifestyle among 7–17 years old children and adolescents in Lithuania. BMC Public Health.

[25] Khalid S, Williams CM, Reynolds SA (2017). Is there an association between diet and depression in children and adolescents? A systematic review. Brit J Nutr.

[26] Beal SJ, Dorn LD, Sucharew HJ, Sontag-Padilla L, Pabst S, Hillman J (2014). Characterizing the longitudinal relations between depressive and menstrual symptoms in adolescent girls. Psychosom Med.

[27] Graves JK, Hodge C, Jacob E (2016). Depression, anxiety, and quality of life in children and adolescents with sickle cell disease. Pediatr Nurs.

[28] Taha AA, Eisen AM, Abdul-Rahman HQ, Zouros A, Norman S (2020). The moderating role of spirituality on quality of life and depression among adolescents with spina bifida. J Adv Nurs.

[29] Shao M-F, Chou Y-C, Yeh M-Y, Tzeng W-C (2010). Sleep quality and quality of life in female shift-working nurses. J Adv Nurs.

[30] Craner J, Sigmon S, Martinson A, McGillicuddy M (2013). Perceptions of health and somatic sensations in women reporting premenstrual syndrome and premenstrual dysphoric disorder. J N Ment Dis.

[31] Karlsson TS, Marions LB, Edlund MG (2014). Heavy menstrual bleeding significantly affects quality of life. Acta Obstet Gyn Scand.

[32] Pinar G, Colak M, Oksuz E (2011). Premenstrual syndrome in Turkish college students and its effects on life quality. Sex Reprod Healthc.

[33] Agarwal K, Agarwal A (2010). A study of dysmenorrhea during menstruation in adolescent girls. Indian J Community Med.

[34] Nevitt J, Hancock GR (2001). Performance of bootstrapping approaches to model test statistics and parameter standard error estimation in structural equation modeling. Struct Equ Model.

[35] Varni JW, Seid M, Kurtin PS (2001). PedsQLTM 4.0: Reliability and validity of the Pediatric Quality of Life InventoryTM version 4.0 Generic Core Scales in healthy and patient populations. Med Care..

[36] Choi ES (2005). Psychometric test of the PedsQLTM 4.0 generic core scale in Korean adolescents. J Nurs Query..

[37] Shin H, Park Y-J, Cho I (2018). Development and psychometric validation of the Menstrual Health Instrument (MHI) for adolescents in Korea. Health Care Women.

[38] Radloff LS (1977). The CES-D scale: A self-report depression scale for research in the general population. Appl Psych Meas.

[39] Chon KK, Rhee MK (1992). Preliminary development of Korean version of CES-D. Korean J Clin Psychol.

[40] Sohn SI, Kim DH, Lee MY, Cho YW (2012). The reliability and validity of the Korean version of the Pittsburgh Sleep Quality Index. Sleep Breath.

[41] Buysse DJ, Reynolds CF, Monk TH, Berman SR, Kupfer DJ (1989). The Pittsburgh Sleep Quality Index: a new instrument for psychiatric practice and research. Psychiat Res.

[42] Korean Youth Risk Behavior Web-Based Survey. https://www.kdca.go.kr/yhs/home.jsp. Accessed 7 Oct 2015.

[43] Han MH, Yoo AJ (1996). The relation of stress and perceived social support to problem behavior. Korean J Child stud.

[44] Lance CE, Beck SS, Fan Y, Carter NT (2016). A taxonomy of path-related goodness-of-fit indices and recommended criterion values. Psychol Methods.

[45] Hsu M-C, Tu C-H (2013). Improving quality-of-life outcomes for patients with cancer though mediating effects of depressive symptoms and functional status: a three-path mediation model. J Clin Nurs.

[46] Choi H, Kim C, Ko H, Park CG (2020). Relationship between sedentary time and sleep duration among Korean adolescents. J Sch Nurs.

[47] Bao Z, Chen C, Zhang W, Zhu J, Jiang Y, Lai X (2016). Family economic hardship and Chinese adolescents' sleep quality: a moderated mediation model involving perceived economic discrimination and coping strategy. J Adolesc.

[48] Baker FC, Kahan TL, Trinder J, Colrain IM (2007). Sleep quality and the sleep electroencephalogram in women with severe premenstrual syndrome. Sleep.

[49] Bourre JM (2006). Effects of nutrients (in food) on the structure and function of the nervous system: update on dietary requirements for brain. Part1: micronutrients. J Nutr Health Aging..

[50] Weng T-T, Hao J-H, Qian Q-W, Cao H, Fu J-L, Sun Y (2011). Is there any relationship between dietary patterns and depression and anxiety in Chinese adolescents?. Public Health Nutr.

[51] American Academy of Pediatrics and American College of Obstetricians and Gynecologists (2006). Menstruation in girls and adolescents: using the menstrual cycle as a vital sign. Pediatrics.

[52] Strotmeyer ES, Steenkiste AR, Foley TP, Berga S, Dorman JS (2003). Menstrual cycle differences between women with type 1 diabetes and women without diabetes. Diabetes Care.

[53] Park Y-J, Ryu H, Han K, Kwon J-H, Kim H-K, Kang H-C (2010). Suicidal ideation in adolescents: an explanatory model using LISREL. Western J Nurs Res.

